# Metabolic signatures and a diagnostic model for citrin deficiency based on urinary organic acids

**DOI:** 10.1002/ctm2.70467

**Published:** 2025-09-14

**Authors:** Peiyao Wang, Peichun Chen, Xinjie Yang, Ziyan Cen, Yu Zhang, Qimin He, Benqing Wu, Xinwen Huang

**Affiliations:** ^1^ Department of Genetics and Metabolism Children's Hospital of Zhejiang University School of Medicine National Clinical Research Center for Child Health Hangzhou China; ^2^ Department of Preventive Health Care Shenzhen Guangming Maternity and Child Care Hospital Shenzhen China; ^3^ Research and Development Center Zhejiang Biosan Biochemical Technologies Co., Ltd. Hangzhou China; ^4^ School of Geography Science and Geomatics Engineering Suzhou University of Science and Technology Suzhou China; ^5^ Research and Development Department Suzhou Bohe Intelligent Information Technology Co., Ltd Suzhou China; ^6^ Department of Neonatology Children's Medical Center, Shenzhen Guangming District People's Hospital Shenzhen Guangdong China

**Keywords:** citrin deficiency, diagnostic model, machine learning, metabolomics, urine organic acids

## Abstract

**Purpose:**

This study aimed to characterise urinary organic acid profiles in Neonatal Intrahepatic Cholestasis caused by Citrin Deficiency (NICCD) and develop a diagnosis model to distinguish NICCD patients from those in the non‐specific metabolic abnormalities group (NAG), both of which exhibit elevated urinary 4‐hydroxyphenyllactic acid (4‐HPLA) and 4‐hydroxyphenylpyruvic acid (4‐HPPA), potentially leading to misdiagnosis.

**Methods:**

A retrospective study was conducted from February 2021 to February 2025, enrolling 105 NICCD patients, 144 healthy controls (HC), and 298 individuals from NAG. Urine organic acids were measured using gas chromatography‐mass spectrometry. Data from NICCD and NAG collected before October 2024 were used for model training and internal testing, with later data serving as an external validation. A three‐step feature selection strategy identified biomarkers. Five machine learning (ML) methods were used to construct the model. Performance was compared using the area under the receiver operating characteristic curve (AUC), accuracy, sensitivity, specificity, *F*1 score, etc.

**Results:**

Compared to HC, NICCD patients exhibited 39 differential metabolites, enriched in tyrosine, aspartate, pyruvate, lipoic acid, and TCA cycle pathways. 4‐HPLA, 4‐HPPA, galactitol, 4‐hydroxyphenylacetic acid, pyruvic acid, quinolinic acid, homovanillic acid, 4‐hydroxybenzoic acid, and malic acid showed high diagnostic performance (AUC > .8). Nine robust markers were identified between NICCD and NAG. The random forest model demonstrated superior classification performance, with high AUC, accuracy, *F*1 score, and low Brier score. An online calculator was developed for clinical use.

**Conclusion:**

Our findings highlight NICCD metabolic enrichment in energy and amino acid pathways and present an interpretable ML model for distinguishing NICCD from those of NAG.

## Graphical abstract

- Urinary organic acid profiles in NICCD show enrichment in energy and amino acid pathways.
- The random forest model differentiates NICCD from non‐specific metabolic abnormalities, both with elevated 4‐hydroxyphenyllactic acid and 4‐hydroxyphenylpyruvic acid, which may cause misdiagnosis.
- Model explanation and web application help physicians make clinical decisions for NICCD diagnosis.

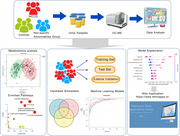

## 1 INTRODUCTION

Citrin deficiency (CD) is now recognised as an autosomal recessive disorder caused by mutations in the *SLC25A13* gene, which encodes citrin, a protein located in the inner mitochondrial membrane. Citrin functions as a key component of the malate‐aspartate shuttle and plays a crucial role in multiple biochemical pathways, including glycolysis, gluconeogenesis, de novo lipogenesis and β‐oxidation, the tricarboxylic acid (TCA) cycle, and the urea cycle.^1^ This disease presents a spectrum of age‐dependent clinical phenotypes, including neonatal intrahepatic cholestasis caused by citrin deficiency (NICCD), failure to thrive and dyslipidaemia caused by citrin deficiency (FTTDCD), and adolescent and adult citrin deficiency (AACD).^2^, ^3^ The clinical features of NICCD typically include intrahepatic cholestasis and a range of metabolic abnormalities such as elevated citrulline levels, often accompanied by increased concentrations of threonine, methionine, and tyrosine, as well as hypoproteinemia, galactosemia, and hypoglycaemia. FTTDCD is characterised by failure to thrive and dyslipidaemia. Approximately 20% of patients may later develop a severe or even fatal metabolic condition known as AACD, characterised by citrullinemia, hyperammonemia, liver steatosis, and cognitive impairment.^4^ Owing to the heterogeneity of clinical manifestations and their overlap with other hepatic and metabolic disorders, the establishment of definitive clinical diagnostic criteria for CD remains challenging.^5^

Early identification of CD is crucial for initiating timely therapeutic interventions that can prevent or mitigate metabolic crises and significantly improve clinical outcomes.^6^, ^7^ Considering the high rate of missed NICCD due to the delayed elevation of plasma citrulline levels,^7^ and the limitations of genetic testing in terms of cost and accessibility, urine‐based metabolite analysis provides a noninvasive and convenient alternative, especially in settings with limited resources or where rapid initial screening is needed. Gas chromatography‐mass spectrometry (GC‐MS), known for its high sensitivity, resolution, and reproducibility, has proven to be a valuable tool for urinary metabolomic profiling.^8^ Although GC‐MS is primarily utilised in the diagnosis of organic acidurias, it can also provide supportive diagnostic information for disorders of amino acid metabolism.^9^ In CD, 4‐hydroxyphenyllactic acid (4‐HPLA) and 4‐hydroxyphenylpyruvic acid (4‐HPPA) have been identified as urinary markers in CD.^10^ However, existing research has largely focused on abnormalities in blood amino acids and acylcarnitines, whereas comprehensive profiling of urinary organic acids in CD remains limited. Moreover, the pathophysiological significance of these urinary alterations is yet to be fully elucidated.

Elevated urinary levels of 4‑HPLA and 4‑HPPA are not specific to citrin deficiency. Similar elevations have been reported in various inherited metabolic disorders, such as tyrosinemia, phenylketonuria, and others.^11^, ^12^ Moreover, these abnormalities may also be observed in patients with parenchymatous liver diseases resulting from infections, tumours, toxins, or nutritional deficiencies such as vitamin C deficiency.^13^ However, some inherited metabolic disorders that exhibit elevated 4‐HPLA and 4‐HPPA also possess disease‐specific urinary biomarkers that aid in differential diagnosis. For example, succinylacetone in tyrosinemia type I, methylmalonic acid in methylmalonic acidemia, and phenylpyruvate or phenyllactic acid in hyperphenylalaninemia. The presence of such specific markers enables a clear distinction from CD. In contrast, when elevated 4‐HPLA and 4‐HPPA are present without accompanying disease‐specific metabolites, CD is at risk of being misdiagnosed, particularly in the absence of supporting plasma amino acid profiles. This diagnostic ambiguity highlights the urgent need for a more reliable diagnostic model to accurately differentiate CD from other conditions lacking specific metabolic signatures.

Machine learning (ML) has demonstrated strong performance in clinical prediction tasks.^14^ Unlike traditional statistical methods, ML algorithms can process multiple features simultaneously and uncover complex, nonlinear relationships among them, which is particularly valuable in clinical research where outcomes often result from interactions between diverse biological and environmental factors.^15^ In the field of metabolic disease detection, prior studies have highlighted the potential of ML‐based approaches.^16^ For example, Haomin Li et al. successfully employed ML models to identify 11 types of inborn errors of metabolism using GC‐MS urinary metabolomic data,^17^ demonstrating the feasibility of ML for rare disease screening. Despite these advancements, the limited interpretability of ML models remains a major barrier to their widespread clinical adoption.^18^ This trade‐off between model complexity and transparency has hindered integration into healthcare settings. To address this, SHapley Additive exPlanations (SHAP) has emerged as a widely adopted interpretability method.^19^ SHAP quantifies the contribution of each feature to individual predictions, helping to mitigate the ‘black box’ nature of ML models.

This study was designed to systematically analyse urinary organic acid patterns in individuals with NICCD and to construct a transparent and interpretable ML model for the diagnosis of NICCD using urinary metabolomic data, thereby deepening our understanding of the underlying metabolic disturbances and providing a valuable supplementary tool to enhance diagnostic clarity in clinical practice.

## 2 METHODS

### 2.1 Study subjects

Between February 2021 and February 2025, clinical information and biological samples from individuals suspected of having inherited metabolic disorders (IMDs) were collected from 34 hospitals across China for retrospective analysis at the Children's Hospital, Zhejiang University School of Medicine. Urinary organic acids were analysed using GC‐MS, while serum amino acids and acylcarnitines were measured using liquid chromatography‐tandem mass spectrometry (LC‐MS/MS). All test results were reviewed by IDM experts.

Patients with clinical diagnostic NICCD were identified by experts based on metabolic profiles, including elevated Cit in MS/MS, with or without concurrent abnormalities in Met, Phe, Tyr, or Arg, and increased 4‐HPLA or 4‐HPPA in urine analysis. Patients were excluded if their profiles were suggestive of multiple IMDs or if they had been diagnosed and treated for NICCD during the recovery or follow‐up phase. A total of 105 clinical testing NICCD patients were enrolled. Age‐ and sex‐matched healthy controls (HC), with no abnormalities in GC‐MS analyses, were included (144 individuals). Individuals were excluded if they had a confirmed or history of IMD or were undergoing treatment or in recovery. Those with elevated 4‐HPLA or 4‐HPPA but without disease‐specific markers for known IMDs, whose abnormalities were attributed to non‐specific conditions (e.g., liver dysfunction, infection, malnutrition) were classified as the Non‐specific Abnormalities Group (NAG). Patients were excluded if they had a confirmed IMD diagnosis or if their profiles contained specific IMD biomarkers. A total of 298 patients were included in the NAG group.

The Ethical Committee of Children's Hospital, Zhejiang University School of Medicine, approved this study (reference number: 2021‐IRB‐292). Written consent was obtained from parents for sample collection and data publication.

### 2.2 Measurements of urine organic acids and amino acids

Urine samples (or dried urine filter paper extracts) were thawed at room temperature and vortex‐mixed thoroughly. A 100 µL aliquot of urine was mixed with 20 µL of freshly prepared urease solution and incubated at 37°C for 30 min to hydrolyse urea. Subsequently, 40 µL of internal standard solution and 900 µL of ice‐cold ethanol were added sequentially to precipitate proteins. The mixture was vortexed for 5 s and centrifuged at 14 000 rpm and 4°C for 10 min. The supernatant was transferred to a new tube, and 50 µL of .2% hydroxylamine in ethanol was added and reacted at 60°C for 10 min to form oximes. Samples were then evaporated to dryness under nitrogen at 60°C. After drying, 100 µL of silylation reagent was added, and the reaction was performed at 70°C for 20 min. Following cooling and centrifugation, the clear supernatant was transferred into autosampler vials for GC‐MS analysis.

Derivatised samples were analysed using an Agilent 7890A‐5975C or 7890B‐5977A GC‐MS system, employing tropic acid as an internal standard for quantification. For each target compound, 92 independent calibration curves were established by plotting the ratio of analyte concentration to internal standard concentration on the x‐axis, and the ratio of analyte response to internal standard response on the y‐axis, enabling matrix effect correction and accurate quantification. Detailed methods are available in the Supplementary file.

Amino acid concentrations in serum samples were measured using Liquid Chromatography‐Tandem Mass Spectrometry (LC‐MS/MS) on an API 4500 LC‐MS/MS system (Triple Quad™ 4500MD, AB Sciex, MA, USA), following the laboratory protocol described in Ref. (^20^).

### 2.3 Data processing

Data preprocessing was performed using MetaboAnalyst 6.0 (www.metaboanalyst.ca), including log10 transformation, normalisation, and filtering out metabolites with more than 50% missing values. Missing values were imputed with small constants (half of the minimum positive value). Orthogonal partial least squares discriminant analysis (OPLS‐DA) was used to explore the urinary organic acid profile of NICCD and identify differentially expressed metabolites (DEMs) between the NICCD and HC groups. DEMs were defined as metabolites with a variable importance in projection (VIP) score > 1, fold change (FC) ≥ 1.5 or ≤  .67, and *p* < .05. Pathway enrichment analysis was performed using KEGG (https://www.kegg.jp/) to investigate associated metabolic pathways.

### 2.4 Discriminative variables between NICCD and NAG

To identify robust features, we applied a three‐pronged variable selection strategy: least absolute shrinkage and selection operator (LASSO) regression, support vector machine‐recursive feature elimination (SVM‐RFE), and VIP‐based selection from OPLS‐DA between NICCD and NAG groups. LASSO reduced multicollinearity by shrinking less informative variable coefficients to zero, with the optimal alpha determined via 10‐fold cross‐validation. SVM‐RFE iteratively removed less relevant features to identify the most predictive subset. Variables with a VIP score > 1 in OPLS‐DA were considered significant. The final feature set was derived from the intersection of variables selected by all three methods for consistency and reliability.

### 2.5 Model development and validation

NICCD and NAG samples collected between February 2021 and September 2024 were randomly divided into a training set (70%) and a test set (30%) using stratified sampling. Samples from October 2024 to February 2025 served as external validation. Five ML models, including logistic regression (LR), random forest (RF), K‐Nearest Neighbour (KNN), extreme gradient boosting (XGBoost), and Support Vector Machines (SVM), were implemented to develop diagnostic models for NICCD. Model hyperparameters were optimised using 5‐fold cross‐validation and grid search on the optimal feature subset (Table S5). Each model was retrained on the full training set with selected features and tuned hyperparameters. Performance was assessed on the training, internal test, and external validation sets, with the latter two primarily for model comparison.

### 2.6 Model performance comparison

Model performance was evaluated in terms of discrimination, calibration, and clinical net benefit. Discrimination was assessed using the area under the receiver operating characteristic curve (AUC), accuracy, sensitivity, specificity, precision, and *F*1 score. Calibration was evaluated with calibration plots and Brier scores, where lower values indicate better calibration. Decision curve analysis (DCA) assesses the net clinical benefit across the threshold probabilities. The optimal model was selected based on performance across these metrics in the test and external validation sets to ensure generalisability and robustness.

### 2.7 Model explanation

Interpreting ML models can be challenging due to their complex, non‐linear nature. To enhance interpretability, we employed SHAP, a game theory‐based approach that quantifies each input feature's contribution to predictions. SHAP provides local and global explanations, increasing model transparency and addressing the ‘black box’ limitation of ML algorithms. The final prediction model was deployed as a web‐based calculator using the Shiny framework, allowing users to input clinical features and receive the predicted probability of NICCD for real‐time clinical decision support.

### 2.8 Statistics

Statistical analyses were conducted using SPSS (v26.0), R (v4.4.2), and Python (v3.12.7). Continuous variables were expressed as medians with interquartile ranges, and categorical variables as counts. Group comparisons were conducted using the Mann‐Whitney U test for continuous variables and the chi‐squared test for categorical variables. A two‐tailed *p*‐value < .05 was considered statistically significant.

**FIGURE 1:**
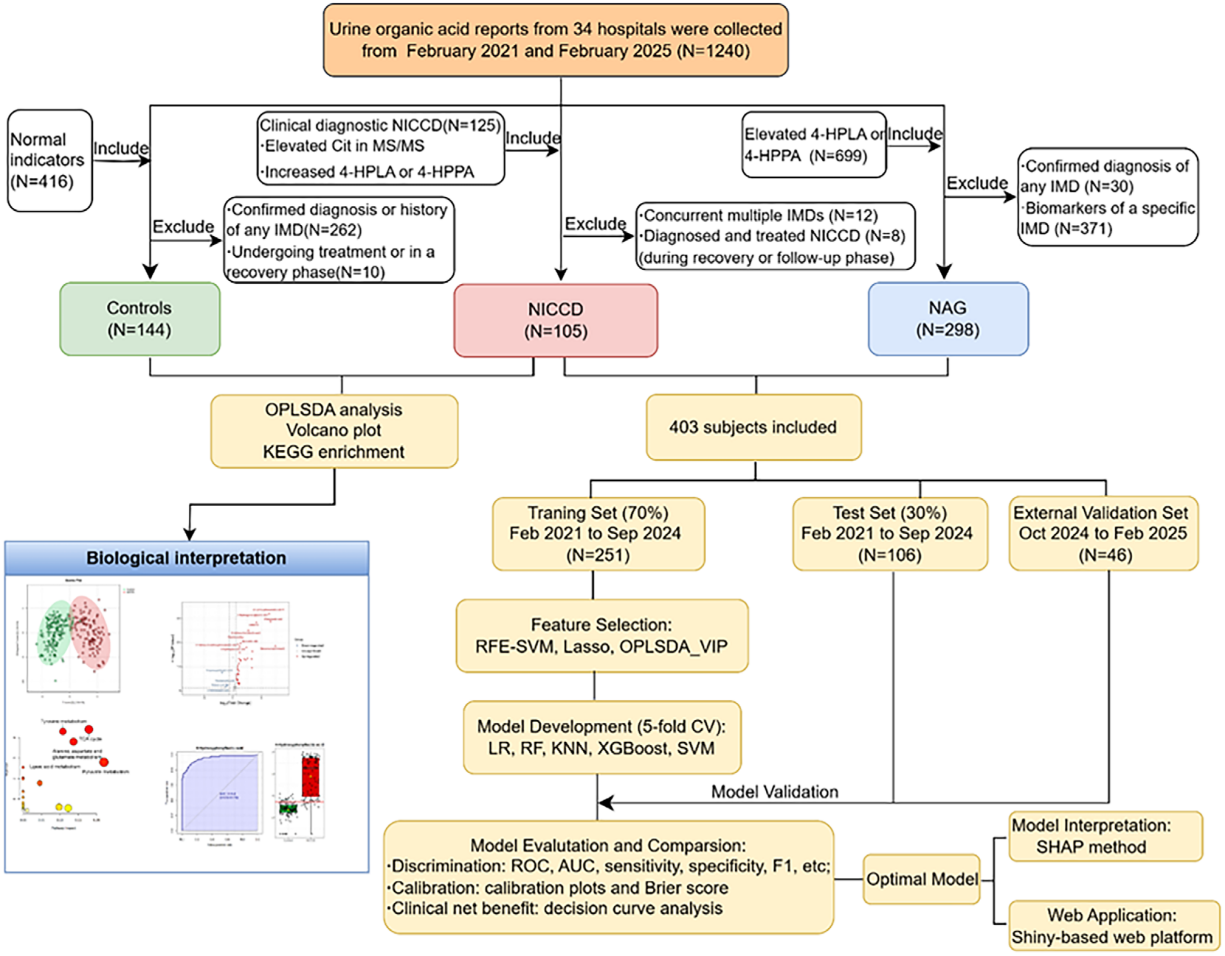
Flow chart of the study design.

The ‘stats’ package was used for the LR model, ‘randomForest’ for RF, ‘class’ for KNN, ‘xgboost’ for XGBoost, and ‘kernlab’ for SVM. Discrimination was assessed by ROC analysis, with AUC and 95% confidence intervals calculated via 1000‐fold bootstrapping. Dose–response relationships were explored using restricted cubic splines (RCS). Study design details are in Figure 1.

## 3 RESULTS

### 3.1 Urinary organic acid profiles in NICCD patients

The NICCD group had a median age of 0 years (IQR: 0–1) with 60 males and 45 females. The HC group had a median age of 0 years (IQR: 0‐0) with 85 males and 59 females. No significant differences were observed between the two groups in age (*p* = .611) or gender (*p* = .766) (Table S1). Of the 92 metabolites analysed, 70 were successfully quantified using GC‐MS/MS. These metabolites were classified into 11 categories, including amino acid metabolism (18.57%), intestinal microbial overgrowth (14.29%), B vitamin group of detoxifications (11.43%), Krebs cycle metabolites (10%), neurotransmitter metabolites (10%) (Figure S1).

**FIGURE 2:**
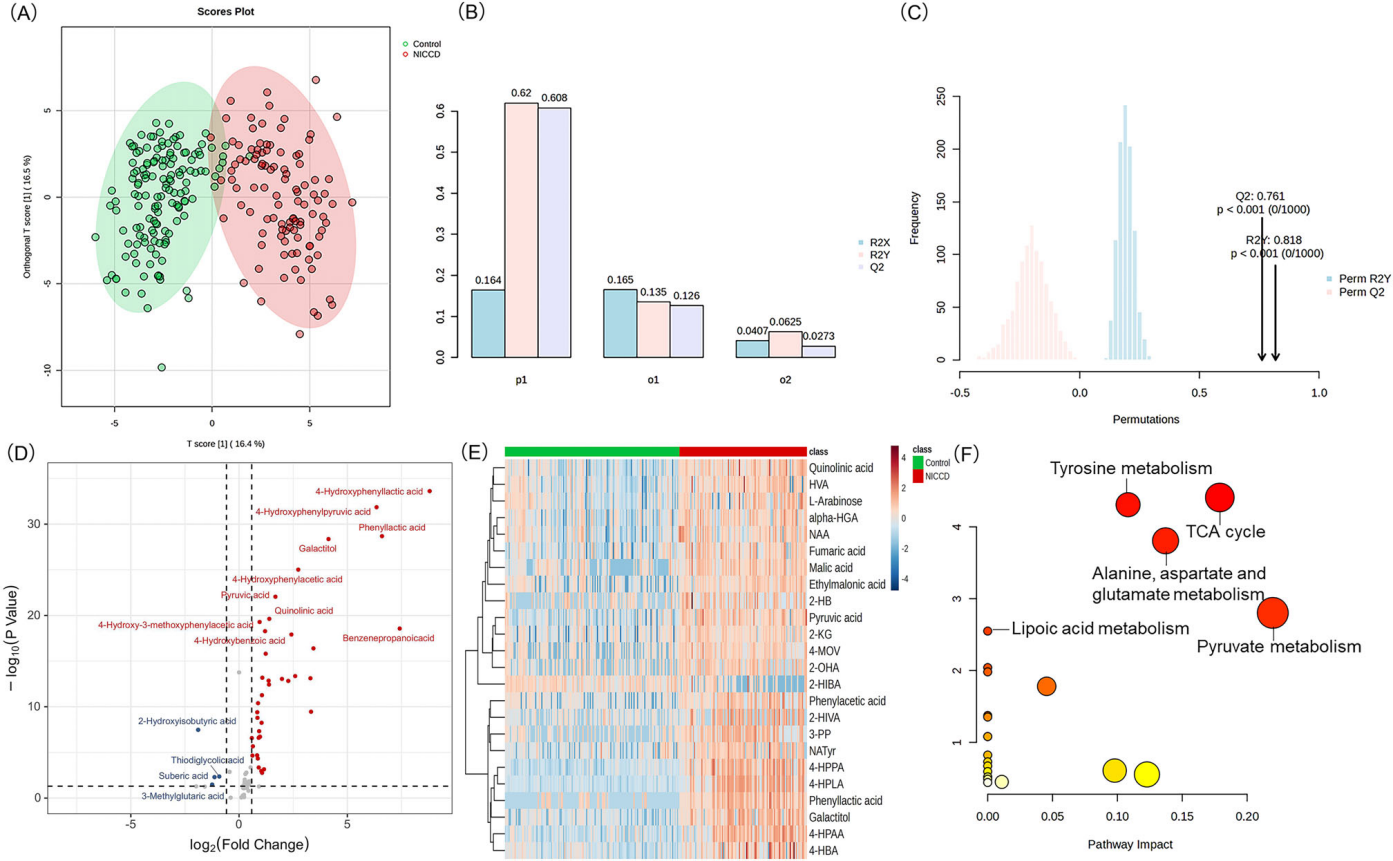
Urinary metabolomic signature in NICCD.

OPLS‐DA revealed a clear separation between NICCD and HC (*R* ^2^ *Y* = .62, *Q* ^2^ = .608), with permutation testing confirming robustness (*p* < .001). The volcano plot (FC = 1.5, *p* < .05) identified 43 DEMs, with 39 upregulated and 4 downregulated. Using VIP > 1, 24 DEMs were identified (Table S2), shown in a hierarchical cluster analysis heatmap. Pathway enrichment analysis highlighted five significant pathways associated with NICCD (FDR < .05): (1) citrate cycle (TCA cycle); (2) tyrosine metabolism; (3) alanine, aspartate and glutamate metabolism; (4) pyruvate metabolism; (5) lipoic acid metabolism (Figures 2 and S2 and Table S3).

### 3.2 Diagnostic performance of urinary organic acids in NICCD

**FIGURE 3:**
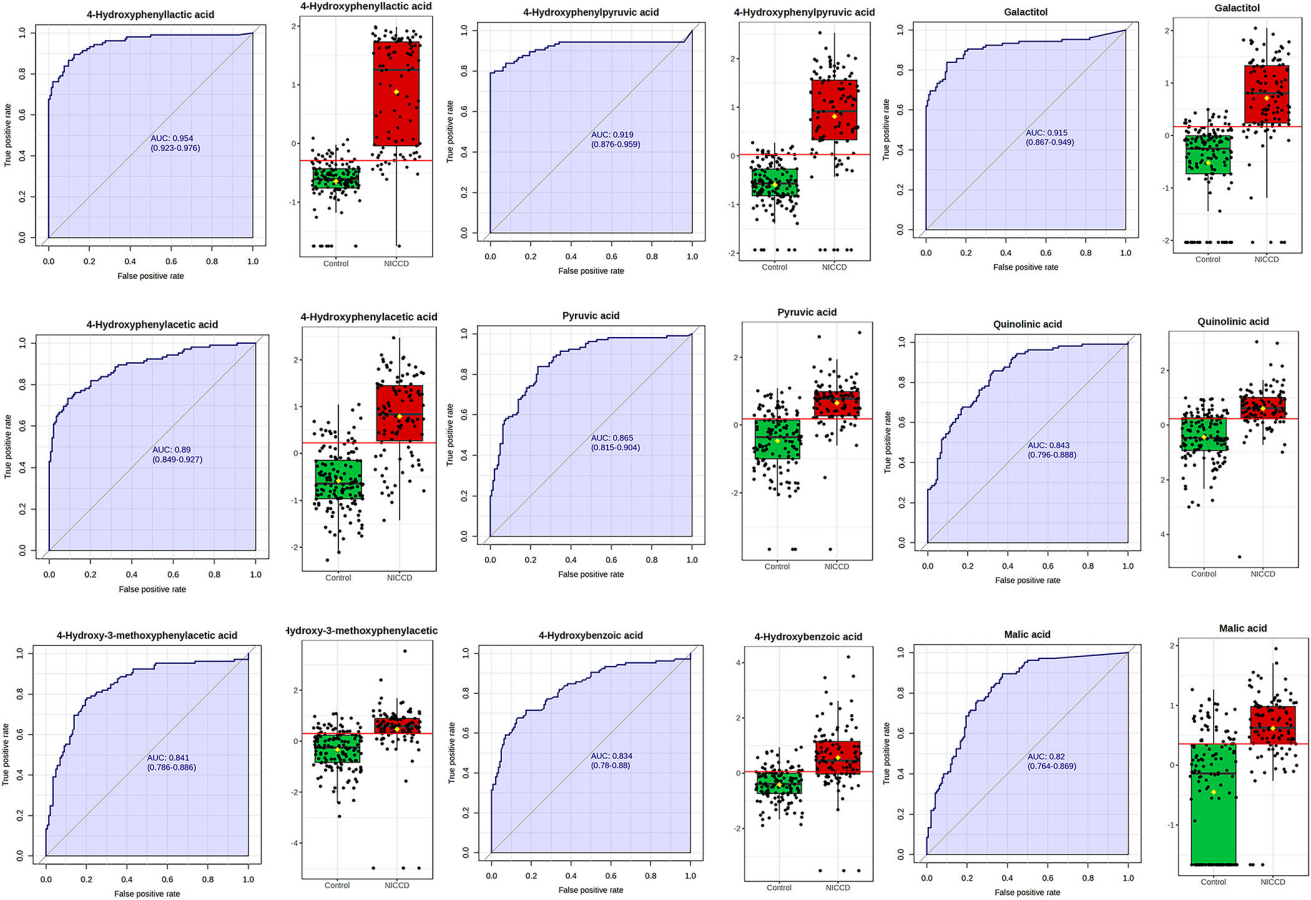
Diagnostic performance of urinary metabolites.

Nine urinary metabolites demonstrated high diagnostic ability (AUC > .8), including 4‐HPLA (AUC = .954, 95% CI:  .925‐.976), 4‐HPPA (AUC = .919, 95% CI:  .869‐.956), and galactitol (AUC = .914, 95% CI:  .871‐.950). Other metabolites like 4‐hydroxyphenylacetic acid (4‐HPAA) (AUC = .890), pyruvic acid (AUC = .865), quinolinic acid (AUC = .843), 4‐hydroxy‐3‐methoxyphenylacetic acid (AUC = .841), 4‐hydroxybenzoic acid (AUC = .834), and malic acid (AUC = .820). All nine metabolites were elevated in the NICCD group compared to the HC (Figure 3).

### 3.3 Distinguishing features between NICCD and NAG

Baseline demographics in the training, test, and external validation are summarised in Table S4. No significant age differences were observed between NICCD and NAG in any cohort (*p* > .05). Sex distribution was comparable between groups in both the training and test cohorts (*p* > .05).

A three‐step feature selection strategy was applied to identify metabolic biomarkers distinguishing NICCD from NAG. First, LASSO regression identified 24 non‐zero coefficient variables. RFE‐SVM with 5‐fold cross‐validation selected 25 features for optimal classification, achieving 87.63% accuracy. OPLS‐DA analysis demonstrated a clear separation between NICCD and NAG (*R* ^2^ *X* = .166, *R* ^2^ *Y* = .527), confirming the model's explanatory and predictive ability. Variables with a VIP > 1 were selected. The final panel of 9 biomarkers (4‐HPLA, galactitol, ethylmalonic acid, phenylacetic acid, glyceric acid, uracil, phenyllactic acid, quinolinic acid, and isoleucine) was identified by the intersection of all three methods (Figure S3).

### 3.4 Dose–response relationship

Nonlinear dose–response relationships between the 9 metabolites and NICCD risk were explored using RCS logistic regression. Age and sex were adjusted for. Significant nonlinear associations were found for 4‐HPLA, galactitol, ethylmalonic acid (EMA), phenylacetic acid, and glyceric acid (*p*‐overall < .05; *p*‐nonlinear < .05). Uracil, phenyllactic acid, quinolinic acid, and isoleucine showed significant overall associations (*p*‐overall < .05) but no nonlinear relationships (*p*‐nonlinear > .05) (Figure S4).

### 3.5 Development and comparison of ML models for differential diagnosis

Five ML algorithms were applied using the nine selected features for differential diagnosis of NICCD and NAG. In the training set, XGBoost performed best, achieving an AUC of  .992 (95% CI:  .985‐.998), followed by RF (AUC = .952, 95% CI:  .922‐.977). In the test set, XGBoost achieved an AUC of  .942 (.889,  .980), accuracy of  .914 (.858,  .962), and *F*1 score of  .833 (.708,  .929). SVM followed closely with an AUC of  .937 (.886‐.978), accuracy of  .848 (.783,  .915), and *F*1 score of  .727 (.583‐.840). RF exhibited stable and competitive performance with an AUC of  .901 (.821,  .960), accuracy of  .878 (.811,  .934), and *F*1 score of  .719 (.558,  .851) (Table S6).

**FIGURE 4:**
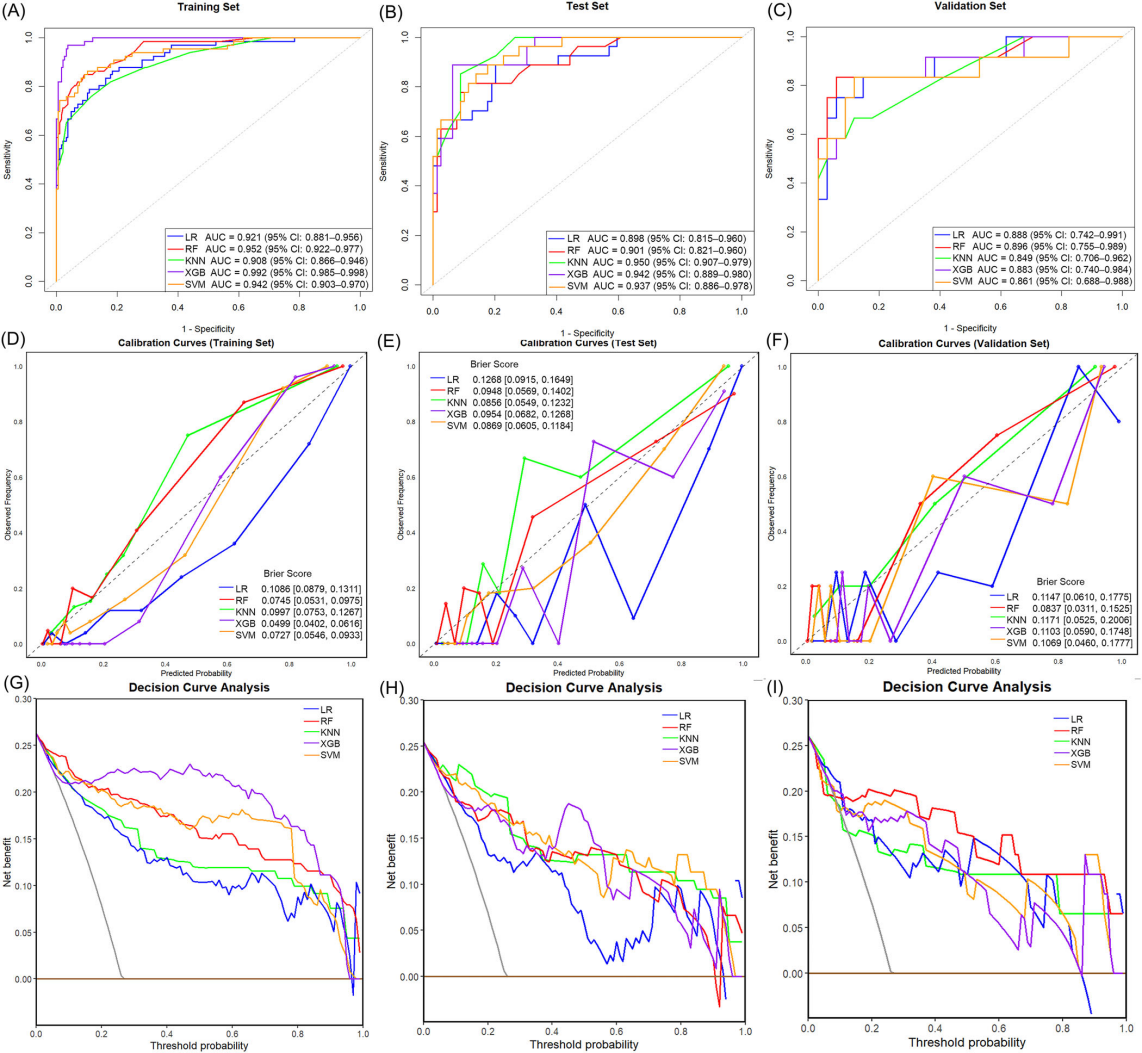
Performance of five ML models across three cohorts.

In the external validation set, which best reflects real‐world generalisability, the RF model outperformed the others, achieving the highest AUC (.892, 95% CI:  .742–.998), accuracy (.892, 95% CI:  .804–.978), and *F*1 score (.757, 95% CI:  .526–.941). RF also demonstrated the smallest AUC difference between test and external validation set (ΔAUC = .009), suggesting strong stability and generalisability compared to XGBoost (ΔAUC = .059) and SVM (ΔAUC = .076). Moreover, RF achieved the lowest Brier score (.0837, 95% CI:  .0311–.1525) and offered the greatest net clinical benefit across a wide range of thresholds in DCA analysis. Based on its consistent performance, generalisability, and calibration, the RF model was selected as the optimal classifier for the differential diagnosis between the two groups (Figure 4).

### 3.6 Model explanation

**FIGURE 5:**
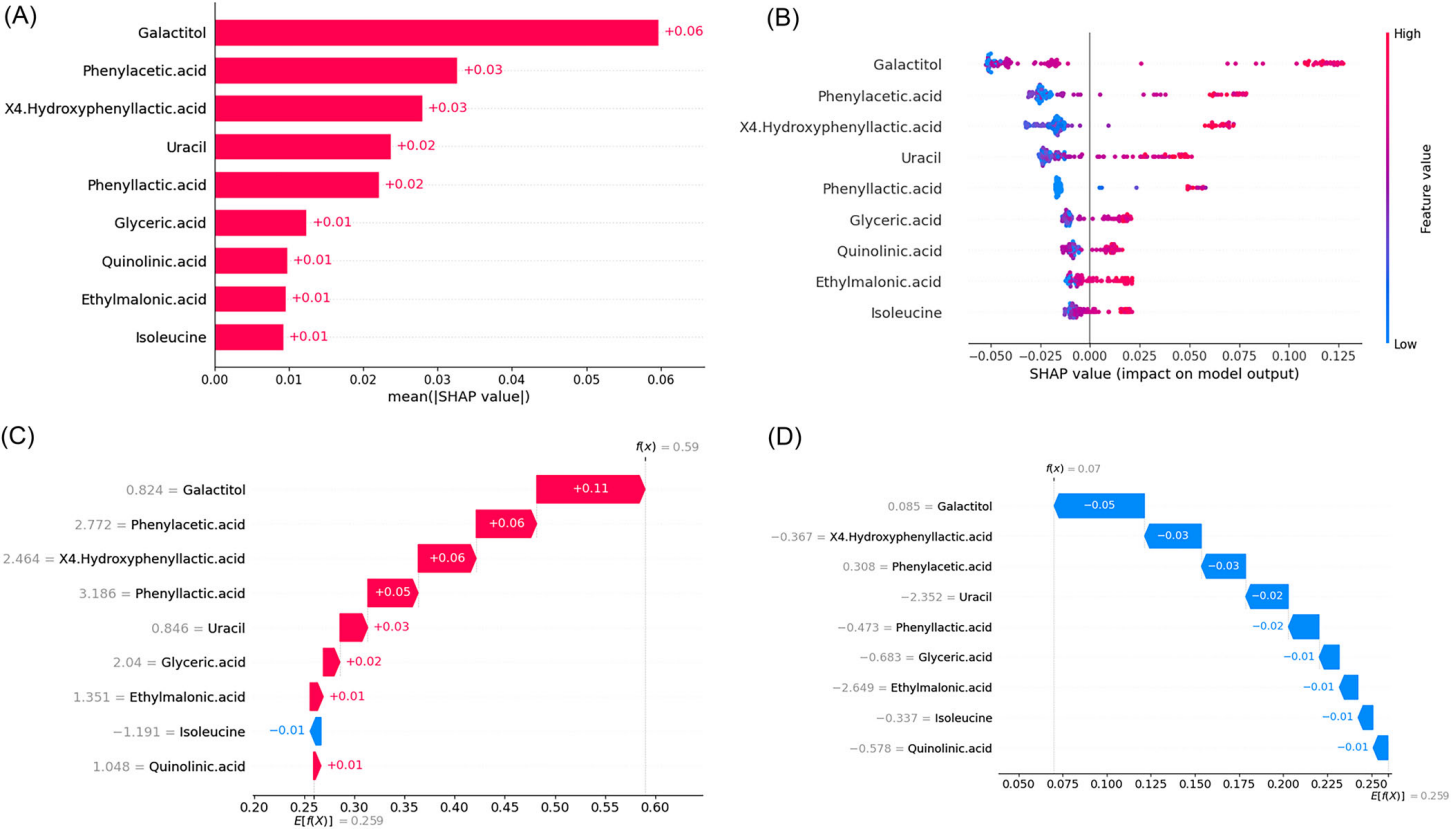
Global and local model explanation by the SHAP method.

**FIGURE 6:**
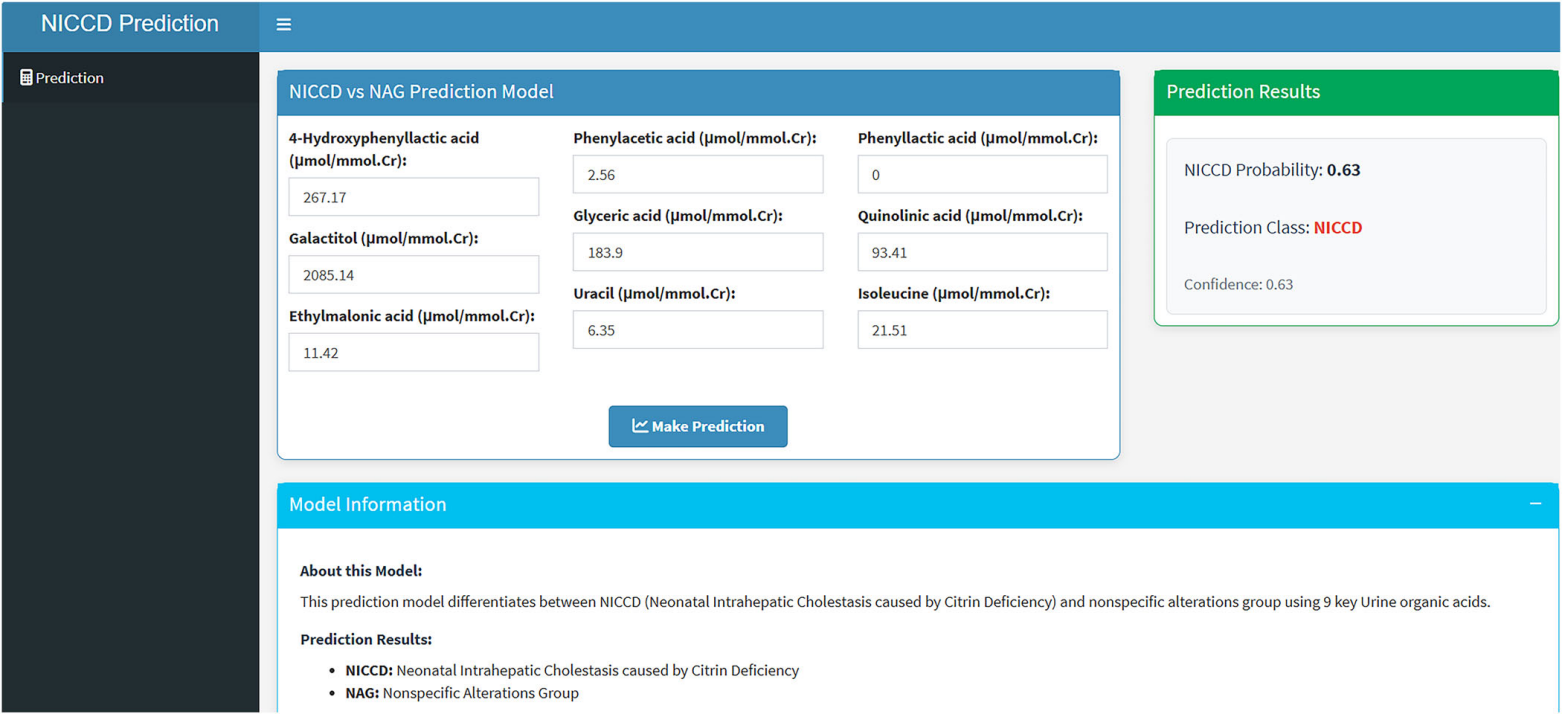
The web‐based calculator for diagnosing NICCD using RF model.

To enhance interpretability, the SHAP method was used to explain the final model's predictions. Global explanations showed the contribution of features to the model, which were displayed in descending order: galactitol, phenylacetic acid, 4‐HPLA, uracil, phenyllactic acid, glyceric acid, quinolinic acid, ethylmalonic acid and isoleucine (Figure 5A). In the SHAP summary dot plot (Figure 5B), the direction and magnitude of each feature's effect on the model are visualised. Higher values of all features were positively associated with NICCD, significantly increasing the probability of diagnosis. Local explanations, using SHAP waterfall plots, show how individual features impact the model's output for specific cases. For instance, Figure 5C shows a representative NICCD case, where galactitol (+.11), phenylacetic acid (+.06), and 4‐HPLA (+.06) were the strongest positive contributors to the model's prediction. Isoleucine had a slight negative impact (‐.01). Other features also contributed to varying degrees. The final model was integrated into a web‐based application for clinical use (Figure 6), allowing users to input values for the 9 features and predict the probability of NICCD. The application can be accessed online at: https://myapp123.shinyapps.io/shinyappniccdvsnag/.

## 4 DISCUSSION

In this study, urinary metabolites in NICCD patients were profiled by GC‐MS/MS, revealing 24 differentially expressed metabolites enriched in energy and amino acid metabolism. Nine showed excellent diagnostic performance (AUC > .8) versus HC. Among five ML models, RF performed best, using a biomarker panel from a three‐step feature selection between NICCD and NAG. SHAP confirmed model transparency, and a web calculator was developed to support clinical application.

Citrin, a liver‐specific aspartate‐glutamate carrier in the inner mitochondrial membrane, is essential for the malate‐aspartate shuttle (MA shuttle), transferring cytosolic NADH into mitochondria to support oxidative phosphorylation and ATP synthesis. Loss of functional citrin reduces mitochondrial NADH, thereby limiting respiratory chain electron donors and impairing energy production. A CE/MS‐based metabolomic analysis of mouse liver extracts revealed that Ctrn‐KO and double‐KO mice exhibited elevated TCA intermediates (e.g., citrate, cis‐aconitate, isocitrate, α‐ketoglutarate, fumarate, and malate) compared to WT and mGPD‐KO mice.^21^ Consistently, our data showed that NICCD patients had increased urinary 2‐oxoglutarate, malate, and fumarate. Tomiko et al. also identified elevated urinary α‐ketoglutarate relative to creatinine as a potential indicator for citrin deficiency.^22^ This accumulation pattern likely results from impairment of the MA shuttle: malate cannot be efficiently transported from the cytosol to mitochondria, leading to cytosolic accumulation and renal excretion. In addition, the elevated cytosolic NADH/NAD⁺ ratio in CD inhibits gluconeogenesis from lactate by limiting the export of mitochondrial malate, thereby causing retention of oxaloacetate and accumulation of TCA cycle intermediates within mitochondria.^21^ The observation that CD patients with normal liver function exhibit increased plasma citrate and α‐ketoglutarate, but decreased fumarate and malate, may indicate that hepatic function may influence TCA metabolite profiles.^23^

Pathway analysis revealed enrichment of lipoic acid metabolism, driven by accumulation of three α‐keto acid metabolites: 2‐oxoadipate, pyruvate, and 2‐oxoglutarate. These substrates undergo mitochondrial oxidative decarboxylation via the 2‐oxoadipate dehydrogenase complex (OADHc), pyruvate dehydrogenase (PDH), and 2‐oxoglutarate dehydrogenase (OGDH), respectively, all of which require lipoic acid, NAD⁺, and other cofactors for catalytic function, forming glutaryl‐CoA, acetyl‐CoA, and succinyl‐CoA, which feed into the TCA cycle.^24^ In CD, cytosolic redox imbalance may secondarily disrupt mitochondrial NAD⁺/NADH homeostasis, impairing the activity of these complexes and leading to substrate accumulation with increased urinary excretion. This pattern reflects a broader mitochondrial enzymatic dysfunction driven by redox dysregulation in CD.

CD reduces cytosolic aspartate, altering serum amino acid metabolism, notably methionine, branched‐chain, and aromatic amino acid (AAA) metabolism.^20^ However, urinary organic acid profiles showed different enrichment. Previous GC‐MS analysis revealed markedly elevated 4‐HPLA, 4‐HPPA, and 4‐HPAA, with mild increases in phenylalanine, phenylacetic acid, and phenylpyruvic acid.^25^ Consistently, our study found significant enrichment in tyrosine metabolism, with prominent increases in urinary 4‐HPLA, 4‐HPPA, 4‐HPAA, and homovanillic acid (HVA). In the catabolic pathway, tyrosine is degraded via 4‐hydroxyphenylpyruvate dioxygenase and fumarylacetoacetase into fumarate and acetoacetate for the TCA cycle. This energy‐generating pathway is primarily localised in the liver. In CD, impaired hepatic function and energy metabolism may reduce these enzyme activities, causing metabolite accumulation (4‐HPLA, 4‐HPPA, and 4‐HPAA) and urinary excretion. HVA, a dopamine metabolism end‐product, is linked to neuropsychiatric conditions such as autism spectrum disorder^26^ and certain forms of depression.^27^ CD is known to present with neurological and behavioural symptoms, including ADHD, inattentiveness, and restlessness, some linked to elevated blood ammonia.^28^ In our study, urinary HVA was elevated in CD patients, suggesting that altered dopamine metabolism may potentially contribute to their neurobehavioural manifestations.

In addition to altered tyrosine metabolism, we observed enrichment of the alanine, aspartate, and glutamate metabolism pathway, with elevated urinary N‐acetyl‐L‐aspartic acid (NAA). NAA is synthesised in neurons and degraded in oligodendrocytes by aspartoacylase.^29^ CD exacerbates oxidative stress,^30^ which impairs oligodendrocyte function and NAA catabolism, leading to its accumulation. Elevated NAA is neurotoxic, promotes demyelination and vacuolisation,^31^ increases cerebral cortex oxidative stress,^32^ and induces neuroexcitation and neurodegeneration,^33^ which may underlie the convulsive seizures seen in CD.

ROC analysis identified 4‐HPLA and 4‐HPPA as top urinary organic acids with excellent discriminatory power (AUC > .9), reinforcing their roles as classical metabolic markers of CD. Galactitol, quinolinic acid, and 4‐hydroxybenzoic acid were also significantly elevated in NICCD with strong diagnostic performance (AUC > .83). CD inhibits UDP‐galactose 4‐epimerase, leading to galactosuria and possibly cataracts.^34^ Elevated quinolinic acid, an excitotoxic metabolite of the kynurenine pathway and N‐methyl‐D‐aspartate receptor agonist, may reflect neuroinflammation and neuronal dysfunction in CD.^35^, ^36^ Elevated 4‑hydroxybenzoic acid, primarily derived from gut microbiota metabolism,^37^ could reflect gut‐liver axis alterations due to cholestasis in CD.^38^

Although urinary 4‐HPLA and 4‐HPPA are recognised as sensitive biomarkers for CD, their diagnostic specificity is limited because elevated levels can also occur in non‐specific conditions such as liver dysfunction, infections, malignancies, intoxications, and malnutrition,^13^ which increases the risk of CD misdiagnosis. Combining urinary organic acids with ML algorithms can improve diagnostic accuracy. Among the models evaluated, XGBoost achieved the best performance in the training and internal test sets but showed a tendency to overfit, limiting its generalisability. In contrast, the RF model demonstrated the most robust and consistent performance, maintaining stable AUC values across both the internal test and external validation sets. In the independent external validation cohort, the RF model outperformed the others by achieving the highest accuracy, superior calibration, and the greatest net clinical benefit, indicating its strong potential for real‐world application. The RF algorithm enhances predictive accuracy and stability by aggregating the outputs of multiple decision trees through a voting mechanism. Its ability to capture nonlinear relationships makes it well‐suited for modelling complex clinical data. Its ensemble approach also reduces the risk of overfitting associated with single decision trees.^39^ RF has been widely recognised as a valuable approach for developing medical prediction models.^40^ In this study, we utilised the RF algorithm to construct a final model comprising nine readily obtainable features, offering a practical and effective tool for the diagnosis of CD.

While including more variables may improve diagnostic power, excessive or irrelevant features can compromise model interpretability and clinical applicability. To address this, we employed a three‐step feature selection strategy combining LASSO regression, RFE‐SVM, and OPLS‐DA, identifying nine discriminative metabolites that consistently distinguished NICCD from NAG. SHAP analysis revealed that higher levels of all nine selected biomarkers increased the likelihood of NICCD. In addition to differentiating NICCD from HC, galactitol and quinolinic acid also distinguished NICCD from NAG in this study, with galactitol being the most significant. 4‐HPLA, phenyllactic acid, and phenylacetic acid are by‐products of alternative pathways in AAA metabolism, formed when primary oxidative routes are disrupted. Specifically, 4‐HPLA derives from 4‐HPPA reduction under impaired tyrosine catabolism,^41^ while phenyllactic acid and phenylacetic acid arise from phenylpyruvate through reductive and oxidative transformations of phenylalanine.^42^ The liver is the primary site for both AAA catabolism and uracil degradation. Higher levels of these metabolites in NICCD than in NAG suggest that cholestasis from citrin deficiency may result in more severe hepatic impairment, leading to greater urinary accumulation and thereby conferring strong discriminative power in distinguishing the two groups. In dried blood spots, Ile+Leu levels were also elevated in NICCD patients,^43^ likely due to impaired glutamine synthetase, which increases their blood and urinary concentrations as precursors of glutamic acid and glutamine.^44^

EMA accumulation occurs in disorders such as short‐chain acyl‐CoA dehydrogenase deficiency, ethylmalonic encephalopathy,^45^ and multiple acyl‐CoA dehydrogenase deficiency,^46^ and is considered a sensitive biomarker of mitochondrial impairment.^47^ In CD, downregulation of peroxisome proliferator‐activated receptor α (PPARα),^48^ a key regulator of mitochondrial fatty acid oxidation, may underlie the higher EMA levels observed compared with NAG. Additionally, PPARα downregulation leads to hypertriglyceridemia.^49^ Impaired MA shuttle activity is partially compensated by the malate‐citrate (MC) shuttle, which promotes fatty acid synthesis.^50^ The glycerol phosphate shuttle also provides an alternative pathway for NADH oxidation, generating glycerol‐3‐phosphate for triglyceride synthesis.^51^ Triglyceride hydrolysis releases glycerol, which is oxidised to glyceric acid.^52^ These lipid metabolism disturbances suggest glycolic acid may help distinguish NICCD from NAG.

ML techniques are often referred to as ‘black boxes’ due to their opaque prediction processes, limiting clinical adoption. A strength of this study is the use of SHAP to provide both global and local explanations, clarifying how patient‐specific data inform predictions. Additionally, using Shinyapps, we deployed the model on an accessible online platform for doctors and patients. Another strength is that the model's diagnostic factors are derived from non‐invasive, easily obtained urine metabolome, supporting clinical applicability. We acknowledge several limitations of the present study. First, the model was developed using data exclusively from Chinese patients, which may limit generalisability without multi‐ethnic validation. Second, the study did not account for socioeconomic or structural factors (such as birth weight and gestational age) that may influence diagnosis. Finally, the study relied on urine metabolome data, which can vary with diet and circadian rhythms.

In conclusion, this study characterises the urinary organic acid profile in NICCD, identifying key mechanisms in energy and amino acid metabolism. We developed an explainable RF model to distinguish citrin deficiency from non‐specific abnormalities with elevated urinary 4‐HPLA and 4‐HPPA, achieving excellent performance in internal and external validations to support clinical diagnosis.

## AUTHOR CONTRIBUTIONS

WPY and CPC should be considered joint first authors. WPY, CPC, and YXJ designed the research, analysed the data, and wrote the manuscript; WPY, CZY, and ZY analysed data and performed bioinformatics analysis; WPY and CPC revised the manuscript; HQM, WBQ, and HXW supervised the research study and should be considered joint corresponding authors. All authors approved the final manuscript to be published.

## CONFLICT OF INTEREST STATEMENT

The authors declare no conflicts of interest.

## ETHICS STATEMENT

This study was approved by the Institutional Review Board of the Ethics Committee in Children's Hospital, Zhejiang University School of Medicine (reference number: 2021‐IRB‐292). Identity information, privacy, and sensitive data of patients were removed before starting the analysis. Therefore, this article did not include privacy data, identity information, or any sensitive data of patients.

## Supporting information



Supporting Information

## Data Availability

Some or all datasets generated during and/or analysed during the current study are not publicly available regarding patient privacy and confidentiality. The data and codes used during the current study are available from the corresponding author upon reasonable request.
