# Supplementary material for: Metabolic signatures and a diagnostic model for citrin deficiency based on urinary organic acids
Source: Clin Transl Med. 2025 Sep 14;15(9):e70467. doi: 10.1002/ctm2.70467 (PMC12434320; doi:10.1002/ctm2.70467)
Supplement: Supplementary file 1 — Supporting Information [file CTM2-15-e70467-s001.docx]

**SUPPLEMENTARY MATERIALS**

1. Methodology

2. Supplementary tables and figures

1. **Methodology**

**Instrumentation and Materials**

The main equipment used in this study included an Agilent 7890A-5975C or 7890B-5977A gas chromatograph–mass spectrometer (Agilent Technologies), a TGL-20MW benchtop high-speed refrigerated centrifuge (Hunan Herexi), a G560E vortex mixer (Scientific Industries), an MD200 heated nitrogen evaporator (Hangzhou Allsheng), a DKS22 thermostatic water bath (Shanghai Jinghong), an AB104-S electronic balance (Mettler Toledo), and Eppendorf micropipettes (100 μL, 200 μL, and 1 mL).

The main consumables included 2 mL, 1.5 mL, and 0.6 mL centrifuge tubes; 200 μL and 1 mL pipette tips; waste liquid containers; 2 mL autosampler vials with 400 μL inserts; and 15 mL sample tubes.

**Reagents**

The reagents used in this study included urease (Sigma, Cat. No. U1500-20KU), analytical-grade ethanol (Hangzhou Longshan), heptadecanoic acid (Sigma, H3500-5G), tetracosane (Aldrich, T8752-25G), tropic acid as internal standard (Alfa Aesar, B22040), HPLC-grade ethyl acetate (Riedel-de Haen), hydroxylamine hydrochloride (Fluka, 55459-50G), and silylation reagent (REGIS BSTFA: TMCS=90:10, Shanghai Anpu). Ultrapure water was produced using a Thermo Scientific GenPure system (CLV-Toc/UF).

**Reagent Preparation**

Urease Solution Preparation: Accurately weigh the required amount of urease powder using an analytical balance and transfer it to a 15 mL centrifuge tube. Add the calculated volume of ultrapure water (required weight × target concentration / 1000), tighten the cap, and gently vortex at low speed until fully dissolved. Avoid vigorous vortexing, ultrasonication, or heating. The prepared urease solution should be stored at 2–8 °C and used within 5 days.

0.2% Hydroxylamine Ethanol Solution Preparation: Accurately weigh 10.0 mg of hydroxylamine hydrochloride into a 25 mL beaker. Add 50 μL of ultrapure water to pre-dissolve, then add 4950 μL of ethanol in portions while mixing thoroughly. Seal the beaker with parafilm after preparation and store at room temperature away from light. The solution should be used within 5 days.

**Experimental procedures**

1. **Urease Treatment**

Urine samples and urease solution were removed from the refrigerator and thawed thoroughly in water at room temperature (approximately 10 minutes in winter or 5 minutes in summer), then vortexed for 30 seconds to mix well. A clean 1.5 mL centrifuge tube was labeled, and 100 μL of urine sample and 20 μL of freshly prepared urease solution were sequentially added using new tips for each step, followed by gentle vortexing for 30 seconds. If the urine creatinine concentration was high, the sample volume or concentration was appropriately adjusted (recommended ranges: 2000–4000 μmol/L for organic acid screening in metabolic disorders, and 4000–6000 μmol/L for nutritional assessments). The mixture was incubated at 37 °C in a water bath for 30 minutes, with the bath preheated in advance when necessary to maintain the target temperature.

1. **Protein Precipitation**

After incubation, 40 μL of internal standard solution was added to each tube (adjusted if needed based on urine creatinine concentration), followed by 900 μL of ice-cold ethanol. The mixture was gently vortexed for 5 seconds and centrifuged at 14,000 rpm and 4 °C for 10 minutes to separate the supernatant. The internal standard solution was pre-thawed and mixed thoroughly before use, and ice-cold ethanol was freshly prepared to ensure optimal cooling. The centrifuge was pre-cooled to 4 °C before operation.

1. **Oximation Reaction**

The collected supernatant was transferred to a clean 2 mL centrifuge tube. Each sample was then mixed with 50 μL of 0.2% hydroxylamine ethanol solution and vortexed gently for 5 seconds. The reaction was performed at 60 °C for 10 minutes in a water bath. After the reaction, the tube cap was opened and the sample was evaporated to dryness in a heated nitrogen evaporator at 60 °C.

1. **Silylation Reaction**

Following evaporation, 100 μL of silylation reagent was added to each tube. The tube was gently tilted to allow the reagent to fully wet and dissolve any residue along the tube wall. The samples were then incubated at 70 °C in a heated nitrogen evaporator for 20 minutes to ensure complete derivatization.

1. **Sample Injection and GC-MS Analysis**

After the reaction, samples were cooled to room temperature and visually inspected. A 100 μL aliquot was transferred to a 0.6 mL centrifuge tube and centrifuged at 14,000 rpm for 4 minutes. The clear supernatant was carefully transferred into autosampler vials equipped with 400 μL inserts, avoiding any pellet while maximizing the transferred volume. The vials were inspected for air bubbles, which were removed if present, before GC-MS analysis and data acquisition.

**Calibration Curve Preparation**

Calibration curves for 92 target compounds were established using seven concentration levels prepared by serial dilution of high-purity stock solutions, covering the expected clinical detection range. A fixed volume of tropic acid internal standard was added to each standard solution, and all standards underwent the same sample preparation steps as urine samples, including urease hydrolysis, protein precipitation, oximation, and silylation derivatization. After GC-MS analysis, peak areas for the analytes and internal standard were obtained, and response ratios were calculated. Calibration curves were generated by plotting the ratio of analyte concentration to internal standard concentration (x-axis) against the ratio of analyte response to internal standard response (y-axis) using least-squares linear regression. Each calibration curve was required to achieve R² ≥ 0.95. Calibration curves were updated weekly and validated in each analytical batch using quality control samples.

**Instrument Quality Control and Data Processing**

All calibration curves were re-established and verified weekly to ensure linearity (R² ≥ 0.95) and quantification accuracy. Each analytical batch included matrix-spiked and quality control samples to monitor instrument performance and method precision. Following GC-MS data acquisition, the method files in Chemstation or MassHunter software were updated with the latest retention times and target/qualifier ion ratios to ensure accurate peak identification. Any peaks with incorrect automated integration were manually reviewed and corrected to maintain quantitative accuracy. All data were processed in batches using Agilent Chemstation or MassHunter software, with automated calculation and reporting of analyte concentrations.

1. **Supplementary figures**


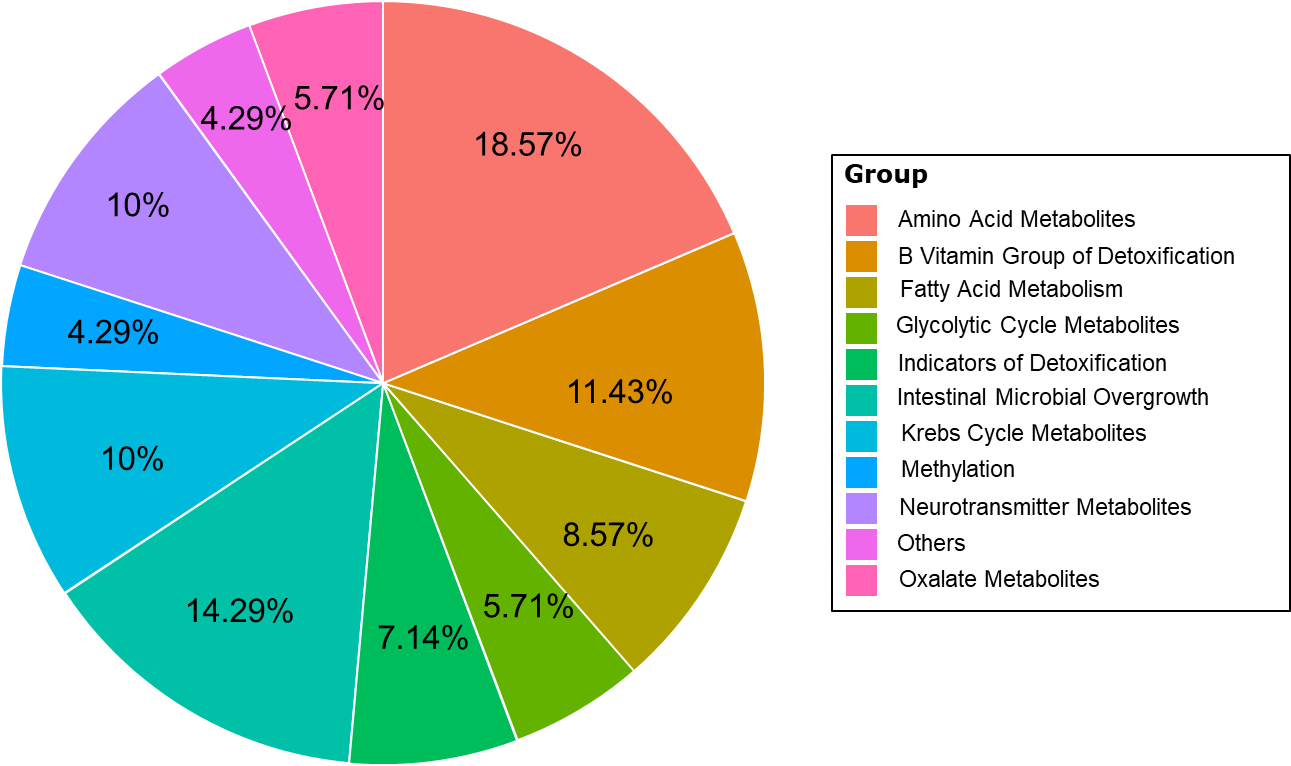


Figure S1. Categories of detected urine metabolites.


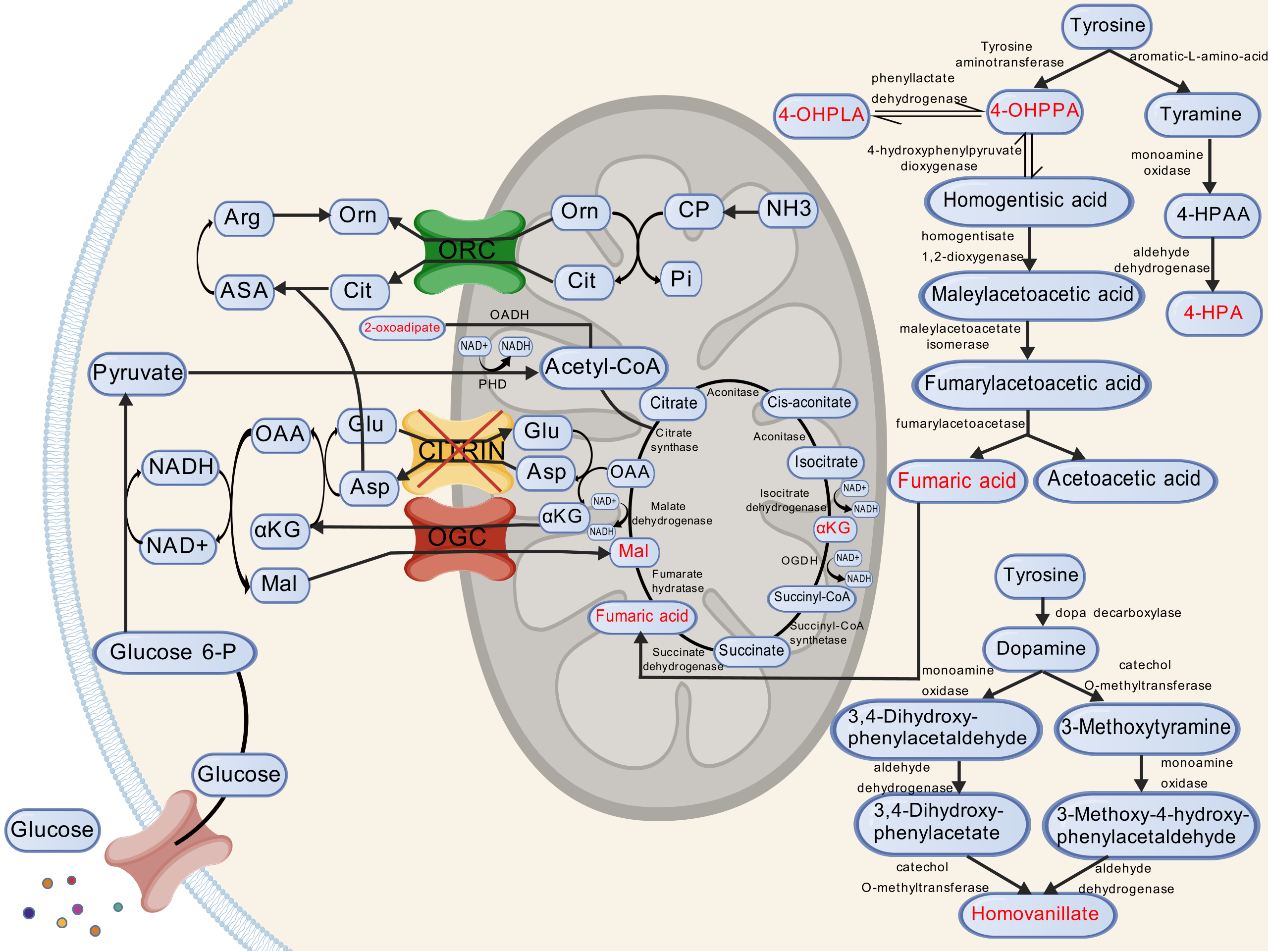


Figure S2. The pathway mapping of the differentially abundant metabolites. Red color indicates the upregulation of DEMs.


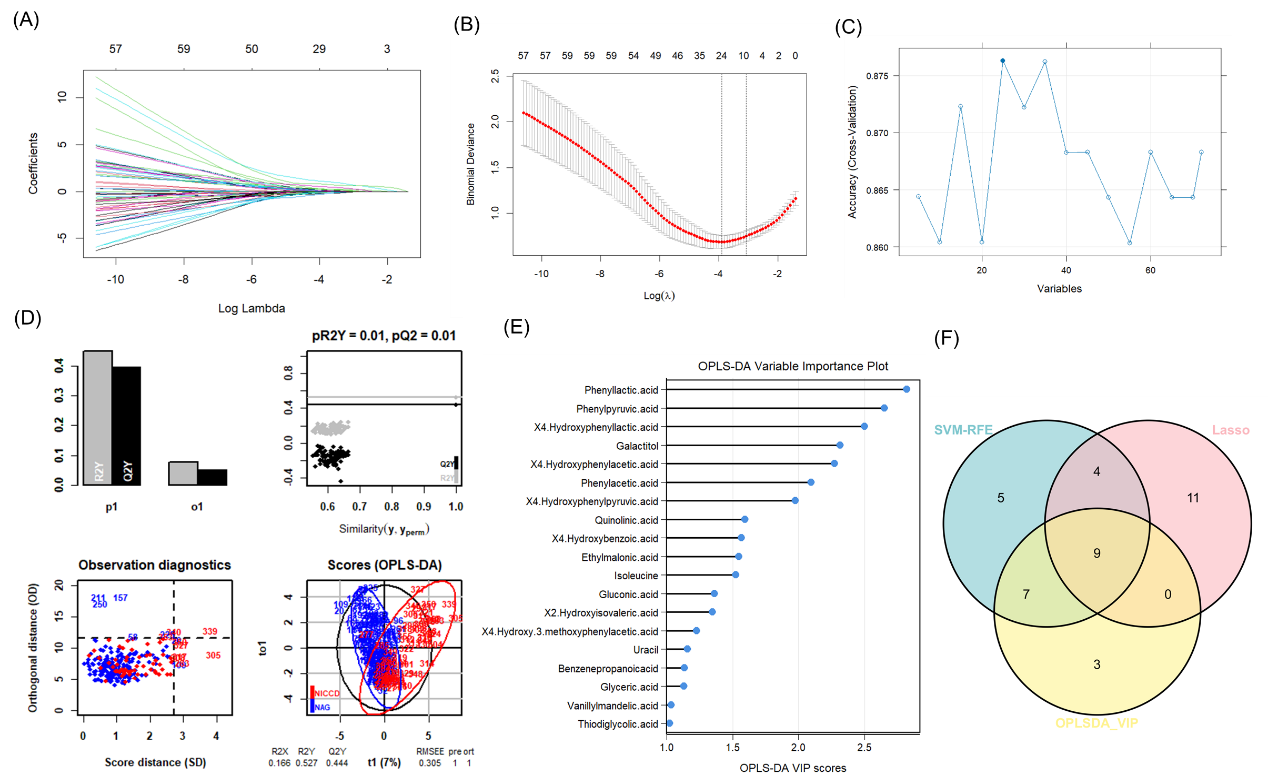


Figure S3. Feature selection results based on three methods. (A) Lasso regression coefficient path plot. (B) Cross-validation error plot. The two dashed lines represent lambda.min(left), the value of lambda that minimizes the cross-validated error, and lambda.1se(right), the largest lambda within one standard error of lambda.min. (C) RFE-SVM identifies the top-ranked metabolites contributing to group classification. (D) OPLSDA model for group separation. (E) VIP>1 were considered significant contributors. (F) Venn diagram summarizing the overlapping selected metabolites from Lasso regression, RFE-SVM, and OPLS-DA methods.


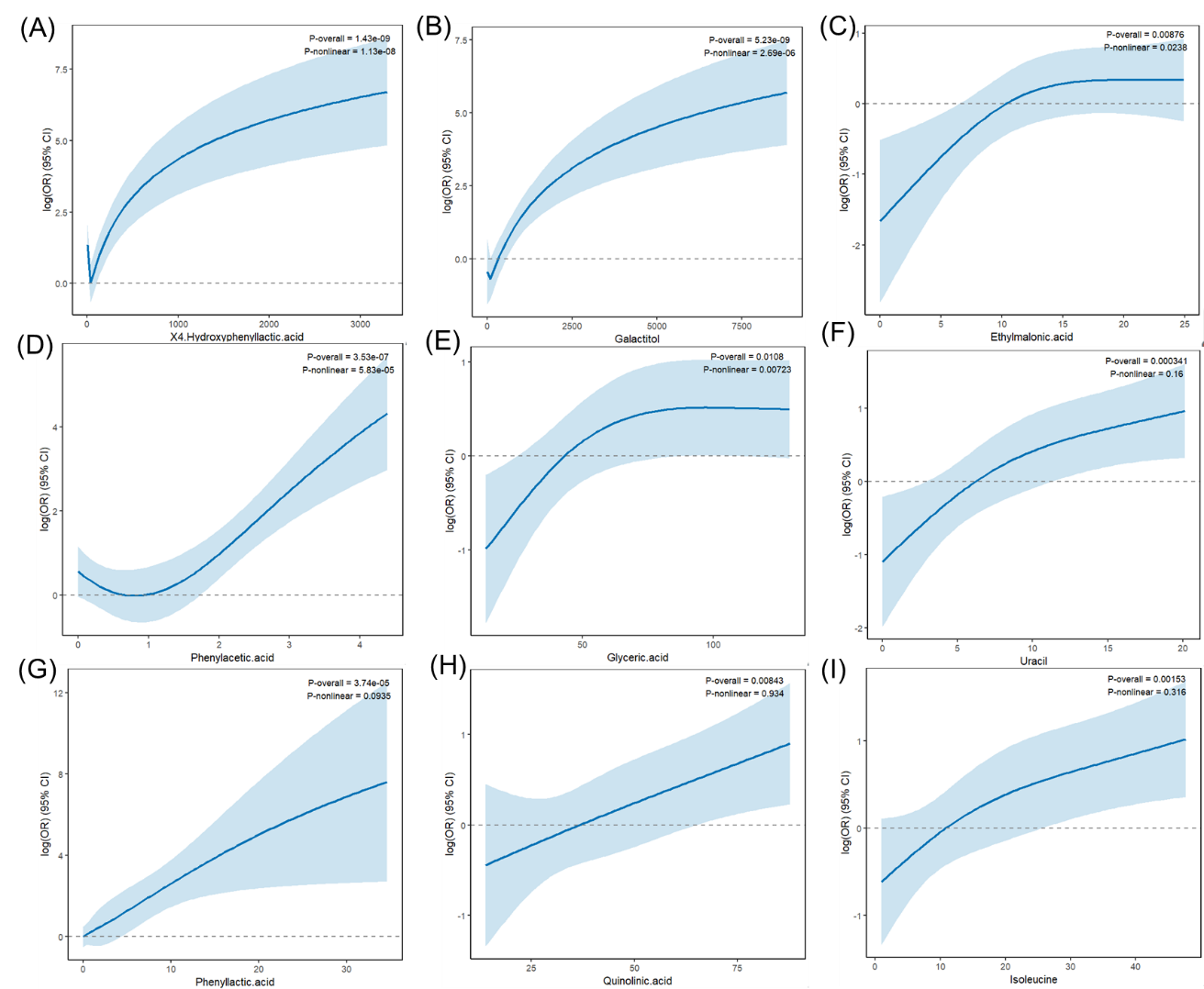


Figure S4. Restricted cubic spline (RCS) plots for different continuous variables. These plots illustrate the nonlinear relationships between each continuous variable and NICCD. The specific continuous variables include 4-HPLA (A), galactitol (B), ethylmalonic acid (C), phenylacetic acid (D), glyceric acid (E), uracil (F), phenyllactic acid (G), quinolinic acid (H), and isoleucine(I). Each variable’s overall and nonlinear relationships are accompanied by P-values, indicating the significance and nonlinearity of the associations.

**3. Supplementary tables**

Table S1. Patients’ characteristics of NICCD and healthy controls.

|  | NICCD(n=105) | HC(n=144) | Z/χ^2^ | P |
| --- | --- | --- | --- | --- |
| Age(year) | 0(0,1) | 0(0,0) | -0.509 | 0.611 |
| Gender(male/female) | 60/45 | 85/59 | 0.089 | 0.766 |

Table S2. Differential Urinary Metabolites Between NICCD and Controls

| Metabolites | VIP | FC | P |
| --- | --- | --- | --- |
| 4-Hydroxyphenyllactic acid | 2.09 | 446.36 | 2.44E-34 |
| 4-Hydroxyphenylpyruvic acid | 2.03 | 81.51 | 1.41E-32 |
| Phenyllactic acid | 1.93 | 97 | 2.08E-29 |
| 4-Hydroxyphenylacetic acid | 1.80 | 6.64 | 9.69E-26 |
| Galactitol | 1.76 | 17.50 | 4.36E-29 |
| Pyruvic acid | 1.58 | 3.20 | 8.91E-23 |
| Quinolinic acid | 1.56 | 2.63 | 2.36E-20 |
| 4-Hydroxybenzoic acid | 1.53 | 2.29 | 5.28E-19 |
| Benzenepropanoicacid | 1.52 | 171 | 2.73E-19 |
| Malic acid | 1.49 | 5.33 | 1.23E-18 |
| Ethylmalonic acid | 1.39 | 2.34 | 1.55E-16 |
| Homovanillic acid | 1.38 | 1.92 | 5.19E-20 |
| 4-Methyl-2-oxovaleric acid | 1.29 | 2.60 | 3.69E-13 |
| Phenylacetic acid | 1.29 | 3.9 | 8.90E-14 |
| N-Acetyl-L-tyrosine | 1.27 | 10.81 | 4.06E-17 |
| alpha-Hydroxyglutaric acid | 1.25 | 2.10 | 6.59E-14 |
| 2-Oxohexanedioic acid | 1.22 | 6.02 | 4.36E-14 |
| Fumaric acid | 1.20 | 2.57 | 1.39E-13 |
| 2-Hydroxybutyric acid | 1.20 | 9.80 | 7.57E-14 |
| 2-Hydroxyisovaleric acid | 1.20 | 10 | 3.52E-10 |
| 2-Ketoglutaric acid | 1.19 | 4.83 | 1.47E-13 |
| 2-Hydroxyisobutyric acid | 1.19 | 0.26 | 3.34E-08 |
| N-Acetyl-L-aspartic acid | 1.08 | 1.83 | 4.05E-11 |
| L-Arabinose | 1.02 | 2.07 | 5.41E-12 |

VIP, variable importance projection; FC, fold change

Table S3. The pathway enrichment analysis.

| Pathway | Total | Hits | FDR | Impact |
| --- | --- | --- | --- | --- |
| Citrate cycle (TCA cycle) | 20 | 2-Oxoglutarate | 0.0019649 | 0.18 |
|  |  | Pyruvate |  |  |
|  |  | Malate |  |  |
|  |  | Fumarate |  |  |
| Tyrosine metabolism | 42 | 4-Hydroxyphenylpyruvic acid | 0.0019649 | 0.11 |
|  |  | 4-Hydroxyphenylacetic acid |  |  |
|  |  | Homovanillate |  |  |
|  |  | Pyruvate |  |  |
|  |  | Fumarate |  |  |
| Alanine, aspartate, and glutamate metabolism | 28 | N-Acetyl-L-aspartate | 0.0041607 | 0.14 |
|  |  | Pyruvate |  |  |
|  |  | Fumarate |  |  |
|  |  | 2-Oxoglutarate |  |  |
| Pyruvate metabolism | 23 | Pyruvate | 0.031426 | 0.22 |
|  |  | Malate |  |  |
|  |  | Fumarate |  |  |
| Lipoic acid metabolism | 28 | 2-Oxoadipate | 0.044987 | 0 |
|  |  | Pyruvate |  |  |
|  |  | 2-Oxoglutarate |  |  |

Table S4. Patients’ characteristics of the NICCD and NAG groups in three sets.

|  | Training Set (70%)  N=251 | |  | Test Set (30%)  N=106 | |  | External Validation Set  N=46 | |  |
| --- | --- | --- | --- | --- | --- | --- | --- | --- | --- |
|  | NICCD  (n=66) | NAG  (n=185) | P | NICCD  (n=27) | NAG  (n=79) | P | NICCD  (n=12) | NAG  (n=34) | P |
| Age(year) | 0(0,0) | 0(0,0) | 0.09 | 0(0,0) | 0(0,1) | 0.15 | 1(1,1) | 1(1,1) | 0.72 |
| Gender  (male/female) | 40/26 | 92/93 | 0.13 | 17/10 | 35/44 | 0.09 | 3/9 | 23/11 | 0.01 |

NAG: Non-specific Abnormality Group

Table S5 Final hyperparameters adopted in the five machine learning models

| ML models | Hyperparameters |
| --- | --- |
| Logistic Regression (LR) | sample weights=inverse frequency |
| Random Forest (RF) | mtry=2 |
|  | ntree=100 |
|  | nodesize=30 |
|  | maxnodes=8 |
|  | sampsize_frac=0.7 |
| KNN | k=19 |
| Xgboost | eta=0.01 |
|  | max_depth=4 |
|  | nrounds=300 |
|  | gamma=1 |
|  | subsample=0.7 |
|  | colsample_bytree=0.6 |
|  | lambda=1 |
|  | alpha=1 |
|  | scale_pos_weight=2.8 |
| SVM | kernel = "rbfdot" |
|  | C = 1 |
|  | sigma = 0.05 |
|  | class.weights= 2.8 |

Table S6. Performance parameters of the five machine learning prediction models

| Cohort | Model | AUC | Accuracy | Sensitivity | Specificity | Precision | F1 score |
| --- | --- | --- | --- | --- | --- | --- | --- |
| Training Set | LR | 0.921  (0.881,0.956) | 0.840  (0.793,0.884) | 0.788  (0.689,0.885) | 0.859  (0.808,0.908) | 0.664  (0.554,0.765) | 0.719  (0.633,0.797) |
|  | RF | 0.952  (0.922,0.977) | 0.889  (0.849,0.924) | 0.605  (0.484,0.718) | 0.989  (0.973,1.000) | 0.953  (0.875,1.000) | 0.738  (0.634,0.852) |
|  | KNN | 0.908  (0.866,0.946) | 0.857  (0.813,0.896) | 0.483  (0.370,0.605) | 0.989  (0.972,1.000) | 0.941  (0.852,1.000) | 0.636  (0.525,0.741) |
|  | XGBoost | 0.992  (0.985,0.998) | 0.961  (0.932,0.984) | 0.940  (0.875,0.986) | 0.968  (0.941,0.990) | 0.913  (0.838,0.973) | 0.926  (0.875,0.966) |
|  | SVM | 0.942  (0.903,0.970) | 0.897  (0.861,0.932) | 0.833  (0.741,0.915) | 0.919  (0.880,0.956) | 0.786  (0.690,0.875) | 0.807  (0.729,0.871) |
| Test Set | LR | 0.898  (0.815,0.960) | 0.801  (0.726,0.877) | 0.776  (0.606,0.923) | 0.810  (0.718,0.894) | 0.583  (0.415,0.737) | 0.662  (0.509,0.789) |
|  | RF | 0.901  (0.821,0.960) | 0.878  (0.811,0.934) | 0.631  (0.440,0.818) | 0.962  (0.911,1.000) | 0.849  (0.682,1.000) | 0.719  (0.558,0.851) |
|  | KNN | 0.950  (0.907,0.979) | 0.878  (0.811,0.934) | 0.521  (0.321,0.714) | 1.000  (1.000,1.000) | 1.000  (1.000,1.000) | 0.680  (0.486,0.833) |
|  | XGBoost | 0.942  (0.889,0.980) | 0.914  (0.858,0.962) | 0.851  (0.714,0.967) | 0.936  (0.883,0.987) | 0.820  (0.667,0.962) | 0.833  (0.708,0.929) |
|  | SVM | 0.937  (0.886,0.978) | 0.848  (0.783,0.915) | 0.812  (0.643,0.950) | 0.860  (0.779,0.932) | 0.665  (0.500,0.815) | 0.727  (0.583,0.840) |
| External Validation Set | LR | 0.888  (0.742,0.991) | 0.847  (0.739,0.935) | 0.749  (0.462,1.000) | 0.883  (0.765,0.973) | 0.697  (0.417,0.933) | 0.713  (0.471,0.900) |
|  | RF | 0.892  (0.742,0.998) | 0.892  (0.804,0.978) | 0.674  (0.400,0.917) | 0.969  (0.900,1.000) | 0.886  (0.625,1.000) | 0.757  (0.526,0.941) |
|  | KNN | 0.849  (0.706,0.962) | 0.846  (0.793,0.935) | 0.418  (0.143,0.714) | 1.000  (1.000-1.000) | 1.000  (1.000-1.000) | 0.578  (0.264,0.833) |
|  | XGBoost | 0.883  (0.740,0.984) | 0.845  (0.739,0.935) | 0.664  (0.400,0.909) | 0.910  (0.811,1.000) | 0.726  (0.416,1.000) | 0.683  (0.444,0.880) |
|  | SVM | 0.861  (0.688,0.988) | 0.848  (0.739,0.935) | 0.668  (0.400,0.909) | 0.913  (0.805,1.000) | 0.736  (0.462,1.000) | 0.691  (0.471,0.880) |
